# Education and micronutrient deficiencies: an ecological study exploring interactions between women’s schooling and children’s micronutrient status

**DOI:** 10.1186/s12889-018-5312-1

**Published:** 2018-04-10

**Authors:** Kassandra L. Harding, Victor M. Aguayo, William A. Masters, Patrick Webb

**Affiliations:** 1 Friedman School of Nutrition Science and Policy, Tufts University, Boston, MA 02111 USA; 2 UNICEF Nutrition Programme, Programme Division, New York, NY USA; 3 Yale School of Public Health, Yale University, 135 College Street, New Haven, CT 06510 USA

**Keywords:** Nutrition, Women’s education, Maternal education, Micronutrients, Micronutrient deficiencies, Economic development, Anemia, Vitamin A, Zinc, Iodine

## Abstract

**Background:**

Formal education can be a nutrition-sensitive intervention that supports the scale-up and impact of nutrition-specific actions. Maternal education has long been linked to child survival, growth, and development while adult earnings and nutrition are tied to years in school as a child. However, less is known about the relationship between maternal education and the micronutrient status of children, women and the general population.

**Methods:**

Using country-level data and an ecological study design, we explored the global associations between women’s educational attainment and: a) anemia and vitamin A deficiency (VAD) in children aged 6–59 months; b) anemia in non-pregnant women; and c) zinc deficiency, urinary iodine excretion (UIE), and the proportion of infants protected against iodine deficiency in the general population Cross-sectional relationships (2005–2013) were assessed using linear regression models.

**Results:**

Percentage of women without schooling was negatively associated with all outcomes. Number of years of schooling among women was positively associated with all outcomes except for UIE and the proportion of infants protected against iodine deficiency. Income level was a significant effect modifier of the effect of years of women’s schooling on child anemia as well as of the proportion of women without formal education on zinc deficiency in the population. The relationship was strongest in low-income countries for child anemia, and was not significant in upper middle-income countries. For zinc deficiency, the relationship was not significant in low or lower middle income countries, which may suggest that a minimum threshold of resources needs to be reached before education can influence zinc status.

**Conclusions:**

While relationships between maternal schooling and micronutrient outcomes vary around the globe, more schooling is generally linked to lower rates of deficiency. These findings draw policy-relevant connections between formal education and anemia and micronutrient status globally. It is necessary to examine the mechanisms through which this relationship may be working at both household and country level.

**Electronic supplementary material:**

The online version of this article (10.1186/s12889-018-5312-1) contains supplementary material, which is available to authorized users.

## Background

Maternal education is an important influence on child survival, growth and development [1, 2]. Education is an underlying determinant of dietary intake and care practices, and, if improved, helps protect children at risk of poor health and nutrition in low- and middle-income countries [3]. For many studies evaluating the relationship between maternal education and child health and nutrition, the main outcome of interest has been either child mortality or child stunting (low height-for-age) [2, 4–12]. For example, in a recent analysis of Demographic and Health Survey (DHS) data from 19 countries, Ruel and Alderman found that the risk of stunting was significantly lower among children of mothers with some primary or secondary education compared to those of mothers with no formal education after controlling for wealth, residence, and child age and sex [2]. In this paper we extend that research to address the association of women’s education with micronutrient deficiencies in children and women, including an assessment of population-level outcomes.

Causality between nutrition and education runs both ways, and both are influenced by national income that facilitates public and private investments in food, health and schooling [13]. Nutrition and education in turn both contribute to higher productivity and earnings, with estimates of undernutrition’s cost ranging from 2.5% of potential national income in India to about 6% in Uganda and over 16% in Ethiopia [14–16]. A significant proportion of the economic costs of undernutrition are due to the effects of poor child nutrition on educational performance and lifelong earnings. That is, impaired child growth and cognitive development due to undernutrition hinders school enrollment at an appropriate age, causes absenteeism or early drop-out due to ill-health or poor learning, and prevents optimal learning and skills development [17, 18].

Education levels have continued to rise in recent decades. The proportion of children up to age 15 worldwide ever attending primary school rose from 81% in 2000 to 85% in 2010, and by 2015 the net enrollment ratio in primary education was estimated at almost 92% of all children [19]. Importantly, the fastest gains have been among girls and women. Between 2000 and 2015, the number of girls for every 100 boys has risen from 92 to 97 in primary education, and from 91 to 97 in secondary education [20]. This change has been accompanied by an even larger decline in the prevalence of stunting, which from 1990 to 2015 is estimated to have declined by about 40% [21]. These promising global trends are driven primarily by rapid success in some regions, as large parts of Africa and Asia continue to suffer from both undernutrition and low school attainment.

Improving nutrition would support improved education outcomes, while enhanced educational performance - particularly of girls, who will become the next generation’s mothers - would help improve intergenerational effects of malnutrition [4, 22, 23]. However, the nature of this bi-directional relationship is not fully understood in terms of sequence, mechanisms, or magnitude of impacts. This is especially true of the links between women’s education and micronutrient deficiencies.

The existing literature focuses heavily on the association between maternal education and child stunting. In this paper, we address the composition of changes differentiated among six indicators of micronutrient deficiencies. Although maternal education is commonly identified as a predictor of anemia, serum retinol, serum zinc, and other measures of micronutrient status among children [24–31], few studies have explicitly compared how maternal education affects each micronutrient deficiency individually. In one study that did look specifically at maternal schooling and maternal nutrition knowledge as determinants of child anemia in Indonesia, both were significant determinants, and maternal nutrition knowledge could substitute for schooling in this relationship [32]. Similarly, another study evaluated maternal education and socioeconomic status (SES) as indicators of anemia in urban Korea and found that maternal education, but not other SES indicators, was inversely related to anemia among 10 year olds [33]. As Block has pointed out, the ability to understand and identify micronutrient content and quality in foods may require more knowledgeable or educated consumers, thus maternal education could be an important determinant of child micronutrient status [32].

This paper seeks to unpack the relationships between maternal education and micronutrient deficiencies and anemia globally, with a specific focus on child outcomes. We focus on women’s educational attainment, with the understanding that this represents the cumulative result of all girls’ education as total number of years spent in school. Where data relate specifically to mothers’ education we use the term ‘maternal education’; otherwise, we apply the term ‘women’s education’. The primary objective of this paper was to define the global associations between women’s education and key micronutrient deficiencies and anemia among children, non-pregnant women, and the general population, describe patterns across time and examine the impact of income on these relationships.

## Methods

We identified recent databases with observations of variables of interest at the national level and globally representative when possible and data access was requested when necessary. We used country-level datasets that included information on educational attainment, gross national income (GNI) per capita, and estimates for anemia and micronutrient deficiencies. Each of the datasets used in this analysis are described below and outcome definitions are summarized in Additional file 1: Table S1.

### Educational attainment

The Barro and Lee educational attainment dataset is a compilation of census data from UNESCO, Eurostat and other sources, first published in 1993. It was most recently revised in 2013, including estimates for each 5 year period between 1950 and 2010, covering 146 countries [19]. For each country and each year, 11 measures of educational attainment data for women, men, and women and men combined are reported. In our analysis, we used 8 such measures: average years of schooling attained in the populations, percentage of no schooling, some primary schooling, completed primary schooling, some secondary schooling, completed secondary schooling, some tertiary schooling, and completed tertiary schooling attained in the population. Based on further evaluation of these variables, we narrowed them down to average years of schooling attained in the populations and percentage of the population without any schooling.

### GNI per capita

World Bank data on GNI per capita in US dollars, converted using the Atlas method, were obtained on October 9, 2015 from the World Bank data website [34]. A total of 249 countries are included in this dataset with GNI per capita data annually from 1960 to 2015. The dataset had no observations for 1960, 1961 and 2015, and included data for 186 countries in 2014. Countries were classified as low, lower middle, upper middle and high income based on the OGHIST analytical income classifications [35].

### Anemia

Stevens et al. (2013) obtained hemoglobin and anemia data for 6–59 month old children and 15–49 year old women from population-representative data sources for 107 countries, from which estimates for hemoglobin distribution and prevalence of anemia among children < 5 y, non-pregnant women and pregnant women for every country for every year between 1990 and 2012 were derived using a Bayesian hierarchical model [36]. We focused our analysis on anemia among children aged 6–59 mo (hemoglobin concentration < 110 g/dL) and non-pregnant women (hemoglobin concentration < 120 g/dL) and applied the WHO definitions to classify countries as having a mild (5–19.9% of the population with anemia), moderate (20–39.9%) or severe (≥40%) public health anemia problem among children < 5 y and/or non-pregnant women [37].

### Vitamin a deficiency

Stevens et al. (2015) compiled data on VAD for 6–59 month old children based on serum retinol concentrations from different population-representative sources that were then used to model prevalence estimates for every year between 1991 to 2013 for 138 low and middle income countries, using a Bayesian hierarchical model [38]. We applied the WHO definitions to classify countries as having a mild (2–9.9% of the population with serum retinol concentrations ≤0.7 μmol/L), moderate (10–19.9%) or severe (≥20%) VAD public health problem among < 5 y [39].

### Zinc deficiency

Wessells and Brown (2012) calculated the average daily per capita availability of major food commodities and then the zinc and phytate content in the foods, by country, using FAO food balance sheet data [40]. Absorbable zinc content was calculated from these values and compared with zinc requirements for each country based on the age and sex distribution to determine estimates of the prevalence of inadequate zinc intake in a population. These country-level estimates were available through open access as supplements to the publication. A total of 210 countries were included in the dataset, 188 of which had data for 1990, 1995, 2000 and 2005.We used the same classification definitions used by Wessells and Brown to identify countries with low (< 15% of the population with inadequate zinc intake), medium (15–25%) or high (> 25%) risk for zinc deficiency.

### Iodine

The Iodine Global Network (formerly ICCIDD Global Network) produced Global Iodine Scorecards in 2012 that compiled data on percent of households consuming iodized salt, median UIE, proportion of the population with UIE less than 100 μg/L, and number of infants and the general population protected and unprotected against iodine deficiency. Inadequate iodine intake is of public health concern when the median UIE is < 100 μg/L and there is risk of excessive iodine intake when UIE is > 300 μg/L among school-aged children in a population [41]. We applied these cut-offs to the country-level median UIE values to classify counties as having inadequate, adequate or excessive iodine levels in the population.

Due to data limitations, an analysis of these relationships is not possible for all micronutrients of interest. While data are available to examine the relationship between women’s education and child vitamin A status, in the case of iron deficiency we use child anemia as a proxy for iron deficiency; in the case of zinc deficiency we use a variable that measures estimated proportion of the population with inadequate zinc intake as opposed to just in children; finally for iodine deficiency we used two variables: the median UIE in the population and the proportion of infants protected against iodine deficiency, derived from data on coverage of households with iodized salt and country birth rates.

As well as assessing the relationship between women’s educational attainment and micronutrient deficiencies, we also determined the nature of these relationships over time, to document if and how they have shifted. While the focus of this analysis was on women’s education, the relationship between men’s education and the outcomes of interest were explored.

### Statistical analysis

Data on adult (both male and female) educational attainment was linked to each nutrition outcome variable and GNI per capita with a 5 year lag period between education indicators (year = n) and the nutrition outcome and GNI per capita data (year = n + 5). To depict the most current relationship between women’s education and micronutrient outcomes, we started with the most recent year for the outcome of interest and combined it with the educational attainment data ≤5 years prior and at least 1 year prior. This exception was made because the educational attainment data was only available for every 5 years (most recent being 2010), whereas most recent data on the outcomes were available for years ranging from 2005 to 2013.

Analysis was conducted using StataSE version 14 (StataCorp). We assessed variables for normality, linear relationships between variables and outliers. Transformations were explored as needed and a log transformation of GNI was included in each model. After exploring different model options, we concluded that linear regression models suit the analysis needs. We used linear regression to evaluate the relationship between educational attainment of women and nutrition outcomes, controlling for country GNI per capita in every model. Because education and GNI are correlated, we examined variance inflation factor (VIF) to test for collinearity in our models. We tested the effect of income classification on the most current relationship between educational attainment of women and outcomes, using an interaction term between income classification and educational attainment. Similarly, we tested whether the relationships differed across time using data from all time points available with the standard 5 year lag between predictor and outcome with an interaction term between year and educational attainment of women and accounted for cluster effect within country. To explore whether the most current relationship between educational attainment and nutrition outcomes differed by adult male or female education, we included both male and female educational attainment in the model, an interaction term between sex and educational attainment, and accounted for clustering within country.

## Results

Sample size and years used for analysis are summarized for each outcome in Table 1. We include analyses on the associations between women’s educational attainment and the following outcomes: a) anemia and vitamin A deficiency (VAD) in children aged 6–59 months; b) anemia in non-pregnant women; and c) zinc deficiency, urinary iodine excretion (UIE), and the proportion of infants protected against iodine deficiency in the general population. Because the analysis comes from various data sources, comparability across outcomes should be done cautiously. The sample of countries for each outcome differs not only in sample size, but also in the distribution across low, lower middle, upper middle, and high income countries. Specifically, 6.4% of countries included in the analysis related to proportion of infants protected from iodine deficiency and 9.7% of countries included in the analysis related to VAD among children aged 6–59 months were classified as high income countries, compared with 28.8% to 36.2% for the analysis of the other outcomes (Table 1).

**Table 1:** Sample size (countries) and year of data used for analysis for all outcomes and distribution of income level of countries by outcome

	Years used to assess current relationship			Years used to assess relationship over time			Income level of countries included in analysis for current relationship [%(n)]			
	Number^a^	Educational attainment	Outcome and GNI per capita	Number^b^	Educational attainment	Outcome and GNI per capita	Low	Lower middle	Upper middle	High
Outcome										
Anemia among < 5 y	141	2010	2012	659	1985–2005	1990–2010	16.3 (23)	24.1 (34)	23.4 (33)	36.2 (51)
Anemia among non-pregnant women	141	2010	2012	659	1985–2005	1990–2010	16.3 (23)	24.1 (34)	23.4 (33)	36.2 (51)
VAD among < 5 y	93	2010	2013	365	1990–2005	1995–2010	23.7 (22)	32.2 (30)	34.4 (32)	9.7 (9)
Zinc deficiency in the population	139	2000	2005	515	1985–2000	1990–2005	25.2 (35)	25.9 (36)	20.1 (28)	28.8 (40)
Median UIE of the population	127	2010	2012	–	–	–	17.3 (22)	26.0 (33)	22.8 (29)	33.9 (43)
Infants protected from iodine deficiency	94	2010	2012	–	–	–	23.4 (22)	36.2 (34)	34.0 (32)	6.4 (6)

^a^ Number of countries

^b^ Number of country time points over the observation periods

*GNI* gross national income, *VAD* vitamin A deficiency, *UIE* urinary iodine excretion, *y* years

Average years of schooling among women across all countries in 2010 (*n* = 146) was 7.6 years and the percentage of women with no schooling was 17.6% (Table 2). Child anemia was classified as being of mild public health significance in roughly 21% (*n* = 30) of countries, while in almost 33% (*n* = 46) it reached levels of severe public health significance. Anemia in non-pregnant women was classified as being of severe public health significance in 13.5% (19) of countries.

**Table 2:** Descriptive statistics

Variable	Number	Mean (SD)
Educational attainment (2010)		
Women’s years of schooling (y)^a^	146	7.6 (2.8)
Men’s years of schooling (y)^a^	146	8.6 (2.1)
Women’s percentage of no schooling (%)^a^	146	17.6 (21.5)
Men’s percentage of no schooling (%)^a^	146	11.9 (15.2)
	year	% (n)
Indicators of micronutrient status across countries		
Anemia among < 5 y (hb concentration < 110 g/dL)	2012	
Mild (prevalence 5.0–19.9%)		21.3 (30)
Moderate (prevalence 20.0–39.9%)		46.1 (65)
Severe (prevalence ≥40.0%)		32.6 (46)
Anemia among non-pregnant women (hb concentration < 120 g/dL)	2012	
Mild (prevalence 5.0–19.9%)		26.2 (37)
Moderate (prevalence 20.0–39.9%)		60.3 (85)
Severe (prevalence ≥40.0%)		13.5 (19)
Vitamin A deficiency among preschool-age children (serum retinol ≤0.70 μmol/l)	2013	
Mild (prevalence 2.0–9.9%)		32.3 (30)
Moderate (prevalence 10.0–19.9%)		25.8 (24)
Severe (prevalence ≥20.0%)		41.9 (39)
Zinc deficiency risk in the population	2005	
Low (< 15% of population at risk for inadequacy)		47.5 (66)
Medium (15–25% of population at risk for inadequacy)		33.8 (47)
High (> 25% of population at risk for inadequacy)		18.1 (34)
Iodine status of countries	2012	
Inadequate (median UIE < 100 μg/l)		20.5 (26)
Adequate (median UIE 100–300 μg/l)		73.2 (93)
Excessive (median UIE > 300 μg/l)		6.3 (8)

^a^ Estimates weighted by population

*hb* hemoglobin, *UIE* urinary iodine excretion, *y* years

VAD was classified as being of severe public health concern in 41.9% (*n* = 39) of the countries in the sample and the risk of zinc deficiency was high in 18.7% (*n* = 26) of the countries included in the analysis related to zinc deficiency. When we applied the cut-offs for adequate, inadequate or excessive iodine levels, iodine range was inadequate in 20.5% (n = 26) of the countries and 6.3% (*n* = 8) of countries were at risk for excessive iodine.

All regression models accounted for GNI per capita, which was significantly associated with the outcomes in all models. We tested for collinearity between GNI per capita and schooling in each model and the results suggested that collinearity was not a problem (VIF < 10). The implication is that while national wealth matters both for investments in educational systems and for building the food and health systems needed to support good nutrition, the degree to which individual girls are able to gain years of education matters independently of wealth.

**Fig. 1:**
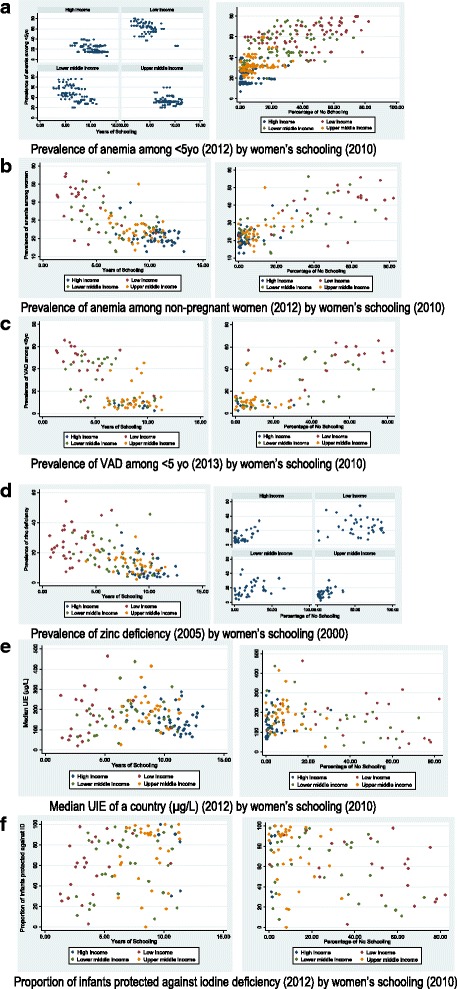
Most recent relationship for nutritional outcomes by women’s years in schooling and percentage of no schooling among countries, by income level. **a** Prevalence of anemia among < 5yo (2012) by women’s schooling (2010). **b** Prevalence of anemia among non-pregnant women (2012) by women’s schooling (2010). **c** Prevalence of VAD among < 5 yo (2013) by women’s schooling (2010). **d** Prevalence of zinc deficiency (2005) by women’s schooling (2000). **e** Median UIE of a country (μg/L) (2012) by women’s schooling (2010). **f** Proportion of infants protected against iodine deficiency (2012) by women’s schooling (2010)

Average years of schooling among women was significantly negatively associated with anemia among children, anemia among non-pregnant women, VAD among children and zinc deficiency among the country’s population, but not with median UIE of the population or the proportion of children protected against iodine deficiency using the most current years available. No schooling for women was significantly associated with all the outcomes evaluated (Fig. 1).

In describing the most current relationships between women’s educational attainment and micronutrient deficiencies, we tested for interactions between country income level and women’s educational attainment for each outcome. Income level was a significant effect modifier for the effect of *years of women’s education* on child anemia, but not on other outcomes. When this model was stratified by the national income levels, women’s years of schooling was no longer associated with child anemia in upper middle income countries (*n* = 33; β(SE) = − 0.34(0.90), *p* = 0.71), but remained a significant predictor of child anemia in all other income categories: low (*n* = 23; β(SE) = − 3.86(1.30), *p* = 0.008); lower middle (*n* = 34; β(SE) = − 2.78(0.98), *p* = 0.008); and high income countries (*n* = 51; β(SE) = − 1.29(0.54), *p* = 0.02)) (Fig. 1a).

When the interaction between *no schooling* for women and income level for each model was tested, we found that the relationship between no schooling in women and zinc deficiency in the population differed by national income level. When this model was stratified by income level, no schooling was no longer a predictor of zinc deficiency among low income (*n* = 35; β(SE) = − 0.05(0.08), *p* = 0.57) or lower middle income countries (*n* = 36; β(SE) = 0.18(0.09), *p* = 0.06), but was a significant predictor among upper middle (*n* = 28; β(SE) = 0.30(0.12), *p* = 0.02) and high income countries (*n* = 40; β(SE) = 0.45(0.09), *p* < 0.001) (Fig. 1d).

For the most recent year, we also considered how male versus female education was associated with anemia and micronutrient outcomes, including both male and female education in a single model and including an interaction term for sex. The average years of schooling among men was significantly associated with all outcomes except with the median UEI and percent of infants protected against iodine deficiency; these relationships did not differ from those of women’s years of schooling. Conversely, the relationship between no schooling among men was significantly stronger for all outcomes except median UIE and percent of infants protected against iodine deficiency, compared with no schooling among women (interaction *p* value between percent of no schooling and sex < 0.05). Results from all models with regards to men’s and women’s educational attainment are presented in Table 3 (Additional file 1: Figure S1).

**Table 3:** Adjusted^a^ beta coefficients of educational attainment indicators for micronutrient deficiencies and anemia

		Average years of school among women				Percentage of no schooling among women			
	n	Beta (SE)	*p* value	Model r^a^	*p* value	Beta (SE)	*p* value	Model r^a^	*p* value
Outcome									
Anemia among children (2012)^b^	141	−2.3 (0.43)	< 0.001	0.75	< 0.001	0.3 (0.05)	< 0.001	0.77	< 0.001
Anemia among non-pregnant women (2012)	141	−1.7 (0.35)	< 0.001	0.46	< 0.001	0.3 (0.04)	< 0.001	0.56	< 0.001
VAD among children (2013)	93	−1.9 (0.75)	0.02	0.58	< 0.001	0.3 (0.08)	0.002	0.6	< 0.001
Zinc deficiency in the population (2005)	139	−0.8 (0.37)	0.03	0.39	< 0.001	0.1 (0.04)	0.04	0.39	< 0.001
Median UIE of the population (2012)	127	−3.0 (4.10)	0.47	0.03	0.24	1.4 (0.50)	0.004	0.09	0.008
Percent of infants protected against iodine deficiency (2012)	94	−3.1 (1.58)	0.05	0.07	0.08	0.5 (0.17)	0.002	0.13	0.006
		Average years of school among men				Percentage of no schooling among men			
	n	Beta (SE)	*p* value	Model r^b^	*p* value	Beta (SE)	*p* value	Model r^b^	*p* value
Outcome									
Anemia among children (2012)	141	−2.1 (0.46)	< 0.001	0.74	< 0.001	0.4 (0.07)^c^	< 0.001	0.76	< 0.001
Anemia among non-pregnant women (2012)	141	−1.2 (0.39)	< 0.001	0.41	< 0.001	0.4 (0.04)^c^	< 0.001	0.56	< 0.001
VAD among children (2013)	93	−1.9 (0.86)	0.03	0.58	< 0.001	0.4 (0.10)^c^	< 0.001	0.61	< 0.001
Zinc deficiency in the population (2005)	139	−1.0 (0.40)	0.02	0.4	< 0.001	0.1 (0.05)^c^	0.01	0.4	< 0.001
Median UIE of the population (2012)	127	−1.1 (4.34)	0.8	0.03	0.29	1.6 (0.66)	0.02	0.07	0.02
Percent of infants protected against iodine deficiency (2012)	94	−1.6 (1.80)	0.39	0.04	0.31	0.5 (0.24)	0.04	0.07	0.07

^a^ All models adjusted for gross national income per capita

^b^ Year of outcome data

^c^ Significantly different from corresponding model with women’s educational attainment

*SE* standard error, *VAD* vitamin A deficiency, *UIE* urinary iodine excretion

**Fig. 2:**
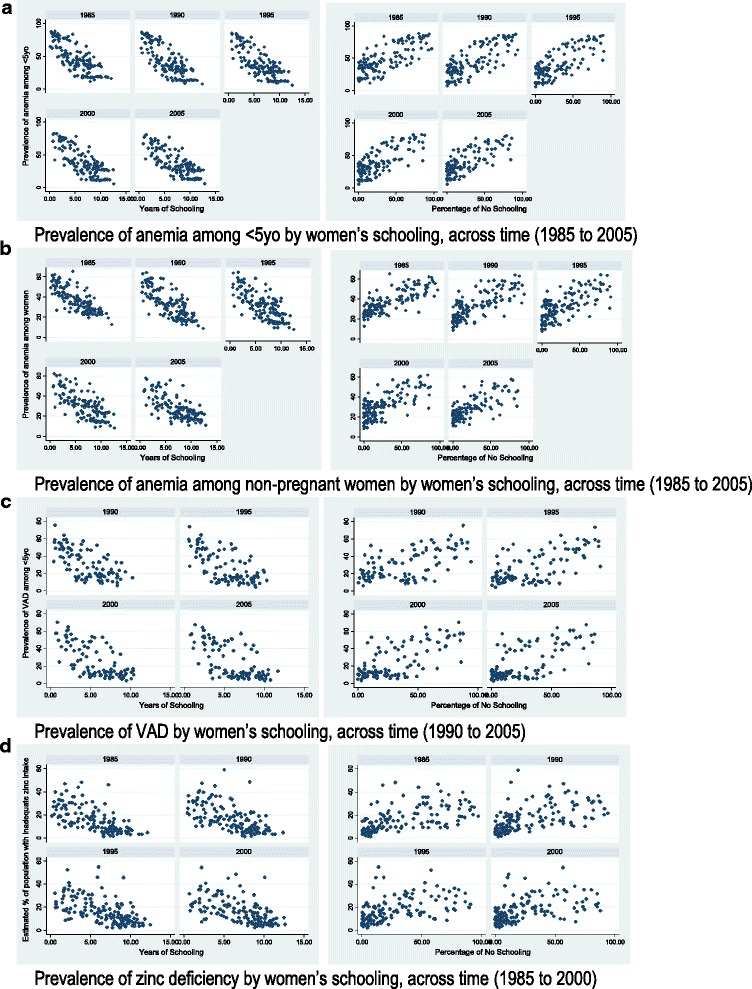
Nutritional outcomes by women’s years in schooling and percentage of no schooling at a country level, across time. **a** Prevalence of anemia among < 5yo by women’s schooling, across time (1985 to 2005). **b** Prevalence of anemia among non-pregnant women by women’s schooling, across time (1985 to 2005). **c** Prevalence of VAD by women’s schooling, across time (1990 to 2005). **d** Prevalence of zinc deficiency by women’s schooling, across time (1985 to 2000)

When trends in the relationships between women’s educational attainment and anemia and micronutrient deficiencies across time were assessed, we found the relationship between the percentage of no schooling among women and the prevalence of anemia among children did not differ significantly, while the relationship between the average years of schooling in women and the percentage of anemic children did differ by year (Fig. 2a). When the model was stratified by year, average years of schooling in women remained significantly negatively associated with anemia in children for all years, though the coefficients and standard errors decline toward zero over time, indicating that the relationship remains precisely estimated by the magnitude of the relationship declines over time (β(SE);P for 1985 = − 3.50(0.59); *p* < 0.001; 1990 = − 3.28(0.48); *p* < 0.001; 1995 = − 2.97(0.45); *p* < 0.001; 2000 = − 2.60(0.46); *p* < 0.001; 2005 = − 2.17(0.42); *p* < 0.001). In the same way, the relationship between women’s educational attainment and the prevalence of anemia in non-pregnant women differed across the years (Fig. 2b). The coefficients for average years of schooling in women ranged from − 2.36 (0.36) in 1985 to − 1.74 (0.34) in 2005, with the magnitude of the relationship decreasing consistently over time while remaining precisely estimated and significant. For the model with percentage of no schooling in women as the main predictor, coefficients increased from 0.25 (0.03) in 1985 to 0.29 (0.04) in 2005, remaining precisely estimated and significant across time.

In the case of both VAD and zinc deficiency, the relationships with women’s educational attainment did not differ by year for either predictor of either outcome (VAD: 1990–2005; zinc: 1985–2000).

## Discussion

Maternal education is known to have a significant impact on child survival and nutrition outcomes including stunting and wasting. In this paper we also found that at a global level adult women’s education is associated with lower anemia and VAD among children, lower anemia among non-pregnant women, and lower zinc in the general population. These are novel findings that underline an additional important reason for investing in getting girls into schools and supporting their full educational attainment [42]. However, the effects are not universal; that is, different relationships are significant for different nutrient deficiencies, suggesting that more than one mechanism (or path for transmitting benefits) are at work. While the mechanism by which maternal education impacts child growth has been extensively studied, little research has been conducted to understand how maternal education can impact children’s micronutrient status. However, we posit that these pathways are likely to overlap, thus we present a number pathways through which women’s education may impact on child nutrition beyond the effects of income, as proposed in the literature. These pathways indicate that women’s education:

1. *Transmits information about maternal and child health and nutrition directly to women, improving mothers awareness and knowledge* [8, 32, 43–47]
2. *Teaches numeracy and literacy, making the acquisition of information more easily attainable* [44, 48]
3. *Ensures exposure to new environments, increasing receptivity to modern health and nutrition knowledge* [44, 49]
4. *Builds greater self-confidence, enhancing women’s roles in decision making and their interactions with health-care professionals* [43, 46] (Though some research indicates this pathway was not present [50, 51]
5. *Provides women with opportunities to form social networks that support women’s empowerment*
6. *Is a proxy for other socioeconomic factors and intergenerational or household patterns* [44, 49, 52, 53]
7. *Delays age of marriage and first child, resulting in fewer and healthier children* [54–56]

Due to the lack of credible and comparable data on covariates within these pathways, this paper was not able to explore which of these, or other, mechanisms may be at work for different nutrients. This is an important knowledge gap given the large ongoing investments by the international community tackle globally-relevant micronutrient deficiencies.

We found evidence of the importance of GNI per capita on the relationship between women’s education and anemia and micronutrient deficiencies globally. Household SES is an important parameter that frames the pathway whereby maternal education can influence children’s nutritional status and the nutritional status of the girl child can influence girl’s education performance. Several studies have shown that children from wealthier families perform better in school, and similarly, higher levels of educational attainment lead to greater income [42]. Using 1998 Bolivia DHS data, Frost et al. found that SES factors were the most important pathways through which maternal education was linked to child nutritional status, though attitudes about modern health care utilization also facilitated this relationship [49]. Yet, the interaction between income and education differs by nutrient of interest. In the case of zinc deficiency, we found that a lack of formal education in women does not predict zinc deficiency in the general population in the low- and lower-middle income countries. This outcome is supported by country-specific studies suggesting that a base level of resources may be required before education can influence nutrition [57, 58]. In other words, education and income may not, in very low income settings, override the fundamental food insufficiency, unsanitary environments and lack of public services that are needed to ensure quality diets and healthcare [59]. Thus, as indicated by the many pathways proposed by the literature, many other things matter in the relationship between women’s education and nutrition beyond wealth.

For example, we may expect that the way in which self-confidence and empowering social networks can improve child growth outcomes (i.e. mothers making decisions about diets and health seeking), would similarly affect micronutrient intakes. The strong links between schooling, delayed marriage, delayed first birth, and fewer, healthier children may impact the micronutrient status of the mother and her children, although this is not a relationship that has been studied as much as effects on child anthropometry and maternal anemia [60].

Similarly, it has been shown that maternal education was associated with adequate compliance with micronutrient supplementation in children aged 3–24 months in Mexico [48] and was associated with the quality of children’s diets (3-day food diaries) in Cyprus [61]. A study in Kenya found that maternal cognitive tests scores - but not maternal education - were positively associated with the quality of the diets of school-age children [62].

There has been some work towards parsing out differences in maternal schooling vis-a-vis health and nutrition knowledge. Block found that in Indonesia, nutrition knowledge and maternal schooling were associated with child anemia and in cases where schooling and income were low, nutrition knowledge was still a determinant of child anemia [32]. Ruel concluded that the effect of maternal schooling on weight-for-age z score was mediated by the nutrition knowledge of the mother in households with access to resources, suggesting that both maternal schooling and the path through which schooling impacts child growth are important [58].

In the case of micronutrient deficiencies, there may be differences in the mechanisms through which maternal education influences each micronutrient outcome, depending on whether knowledge, resources or other constraints impact the acquisition or use of the nutrients, and at what level these constraints work (e.g. individual, household, community, or country). Child vitamin A status, for example, may be influenced by the primary caregiver’s knowledge of and access to high-dose vitamin A supplements, knowledge of vitamin A rich foods, resources to acquire vitamin A rich foods, etc., thus the mechanism through which women’s schooling is associated with child VAD could be through increased knowledge or through increased income and resources that allow for a vitamin A sufficient diet.

Some countries have or are in the process of implementing ‘universal’ supplementation programs as part of health service delivery, and/or establishing regulations for mandatory fortification of target foods with various micronutrients. In such cases, the importance of education to the ability of women to identify and procure specific foods rich in key micronutrients may be lessened, but the value-added of education to micronutrient status would remain, albeit operating via alternative mechanisms. For example, in our analysis, iodine status was not associated with years of women’s schooling, perhaps because in most contexts iodine status is less dependent on dietary choice than on national regulations for universal salt iodization. Once a country successfully reaches the threshold used to define ‘universal iodization’, the consumer does not decide between consuming iodine or not.

Our findings also indicate that years of schooling among adult men showed the same pattern of significant negative associations with anemia and VAD in children, anemia in women, and zinc deficiency in the general population. The significant association between no schooling of men and these outcomes was stronger than that with no schooling of women. It could be that men’s education is working primarily through the mechanism of increased income, which may be less diluted at the population level, whereas women’s education could be influencing micronutrient status through other pathways discussed above. We are not able to tease this out with the current analysis; however, such gendered differences in transmission pathways should be explored further.

While the results presented here are novel and strong, there are a number of limitations to this analysis that should be considered. This was an ecological study designed to quantify relationships at the global level highly informative, it cannot capture the nuances of relationships studied. Additionally, we anticipate variation at the individual level and between countries. In a 19 countries analysis, country-specific regressions indicated that there was diversity among countries in relationships between parental education and child’s height, indicating context specificity of this relationship [4].

Furthermore, we are unable to speak to the effect of an individual mother’s education on her own child’s micronutrient status, which should be a focus of future studies. Much of the data used are derived from modeling, including anemia and VAD in children aged 6–59 months, and anemia in non-pregnant women. Finally, the outcomes of interest were measured using different approaches and each included a number of inbuilt assumptions. For example, VAD, anemia, and UIE were measured using biochemical indicators, but zinc status was based on dietary availability at the country level, and the proportion of infants protected against iodine deficiency was based on a household level diet indicator. As well, we consider anemia as a proxy of iron deficiency, while recognizing that there are a number of nutritional and non-nutritional causes of anemia including non-modifiable factors such as genetic disorders. There remains a critical need for greater data comparability globally, standardization of thresholds used across countries, and disaggregation in many instances by child and adult age and by sex [63].

Despite these limitations, this analysis has a number of important strengths. To our knowledge, it is the first analysis that examines the relationship between women’s education and micronutrient deficiencies and anemia across countries. We account for national per capita income and changes over time, thereby helping to explain how women’s education modifies the relationship between income and malnutrition [64].

## Conclusions

Education, especially women’s education, should remain at the forefront of the global development agenda. Many studies have clearly demonstrated the important relationship between maternal education and children’s survival, growth and development. We add to this body of knowledge by identifying global trends specific to women’s education and micronutrient deficiencies and anemia; we find that as average years of women’s schooling increases, the prevalence of anemia and VAD in children, anemia among non-pregnant women, and zinc deficiency in a country’s general population all decrease. While primary education enrollment is important, this study shows that years of education was associated with micronutrient status in children, women and populations, suggesting the importance of not just enrollment but also of continuation in school. Beyond the clear value of formal education to nutrition, schooling offers an entry point for specific actions that can support nutrition gains more broadly. Educational settings can be used as platforms for nutrition-specific interventions, for example providing nutritious foods at school such as in *Food for Education/School Feeding Programs* [65] or providing iron and folic acid supplements, deworming prophylaxis, and nutrition education as in the Adolescent Girls Anemia Control Programme in India [66]. Enabling and promoting women’s education can enhance the progress made with nutrition-specific interventions via the mechanisms discussed in this paper.

Harnessing efforts to ensuring girls education – especially good quality secondary education - that empowers women to make choices, while scaling-up the many well-documented cost-effective nutrition-specific interventions will accelerate progress towards the global nutrition goals [67] while bringing about great gains in human capital and sustainable development globally.

## Additional file

Additional file 1: **Table S1.** Definitions of the six outcome variables. **Figure S1.** Nutrition outcomes by women and men’s years in schooling and percentage of no schooling at a country level. **A**. Prevalence of anemia among < 5yo (2012) by women and men’s schooling (2010). **B**. Prevalence of anemia among non-pregnant women (2012) by women and men’s schooling (2010). **C**. Prevalence of VAD (2013) by women and men’s schooling (2010). **D**. Prevalence of zinc deficiency (2005) by women and men’s schooling (2000). **E**. Median UIE (μg/L) of a country and proportion of infants protected against iodine deficiency (2012) by women and men’s percentage of no schooling (2010). (DOCX 85 kb)

## Abbreviations

DHS: Demographic and Health Survey
GNI: Gross national income
SES: Socioeconomic status
UIE: Urinary iodine excretion
VAD: Vitamin A deficiency
VIF: Variance inflation factor
