# Supplementary material for: Education and micronutrient deficiencies: an ecological study exploring interactions between women’s schooling and children’s micronutrient status
Source: BMC Public Health. 2018 Apr 10;18:470. doi: 10.1186/s12889-018-5312-1 (PMC5894221; doi:10.1186/s12889-018-5312-1)
Supplement: Supplementary file 1 — Table S1. Definitions of the six outcome variables. Figure S1. Nutrition outcomes by women and men’s years in schooling and percentage of no schooling at a country level. A. Prevalence of anemia among < 5yo (2012) by women and men’s schooling (2010). B. Prevalence of anemia among non-pregnant women (2012) by women and men’s schooling (2010). C. Prevalence of VAD (2013) by women and men’s schooling (2010). D. Prevalence of zinc deficiency (2005) by women and men’s schooling (2000). E. Median UIE (μg/L) of a country and proportion of infants protected against iodine deficiency (2012) by women and men’s percentage of no schooling (2010). (DOCX 85 kb) [file 12889_2018_5312_MOESM1_ESM.docx]

**Additional file 1: Table S1 and Figure S1**

**Table S1**. Definitions of the six outcome variables

| ***Outcome*** | **Country level estimate** | **Population level cut-offs defining public health concern**^1^ |
| --- | --- | --- |
| Anemia among < 5 y | Prevalence of < 5 y with hemoglobin concentration <110 g/dL^2^ | Mild (prevalence 5.0-19.9%)  Moderate (prevalence 20.0-39.9%)  Severe (prevalence ≥40.0%)^2^ |
| Anemia among non-pregnant women | Prevalence of non-pregnant women with hemoglobin concentration <120 g/dL^2^ | Mild (prevalence 5.0-19.9%)  Moderate (prevalence 20.0-39.9%)  Severe (prevalence ≥40.0%)^2^ |
| Vitamin A deficiency among <5 y | Prevalence of < 5 y with serum retinol ≤ 0.70 µmol/l^3^ | Mild (prevalence 2.0-9.9%)  Moderate (prevalence 10.0-19.9%)  Severe (prevalence ≥20.0%)^3^ |
| Zinc deficiency in the population | Percent of population with estimated inadequate intake based on 1) average daily per capita availability of major food commodities 2) zinc and phytate content in the foods 3) zinc requirements based on the age and sex distribution^4^ | Low (<15% of population at risk for inadequacy)  Medium (15-25% of population at risk for inadequacy)  High (>25% of population at risk for inadequacy) ^4^ |
| Median UIE of the population | N/A – median UIE (µg/l) ^5^ | Inadequate (median UIE < 100 µg/l)  Adequate (median UIE 100 – 300 µg/l)  Excessive (median UIE > 300 µg/l) ^5^ |
| Infants protected from iodine deficiency | Percent of infants in a household with iodized salt^5^ | N/A |

UIE, urinary iodine excretion; y, years

^1^Median UIE represents the country level iodine status based on median UIE

^2^UNICEF, UN University, and WHO, *Iron deficiency anaemia: assessment, prevention, and control. A guide for programme managers.*, WHO/NHD/01.3, Editor. 2001, WHO: Geneva, Switzerland.

*^3^Serum retinol concentrations for determining the prevalence of vitamin A deficiency in populations*, WHO/NMH.NHD/MNM/11.3, Editor. 2011: Geneva, World Health Organization.

^4^Wessells, K.R., G.M. Singh, and K.H. Brown, *Estimating the global prevalence of inadequate zinc intake from national food balance sheets: effects of methodological assumptions.* PLoS One, 2012. **7**(11).

^5^WHO, UNICEF, and ICCIDD, *Assessment of iodine deficiency disorders and monitoring their elimination : a guide for programme managers. – 3rd ed.* 2007: Geneva, Wold Health Organization.

**A.** Prevalence of anemia among <5yo (2012) by women and men’s schooling (2010)

**B.** Prevalence of anemia among non-pregnant women (2012) by women and men’s schooling (2010)

**C.** Prevalence of VAD (2013) by women and men’s schooling (2010)

**D.** Prevalence of zinc deficiency (2005) by women and men’s schooling (2000)

**E.** Median UIE (µg/L) of a country and proportion of infants protected against iodine deficiency (2012) by women and men’s percentage of no schooling (2010)

**Figure S1.** Nutrition outcomes by women and men’s years in schooling and percentage of no schooling at a country level
